# Cardiac Rehabilitation in Patients with Implantable Cardioverter-Defibrillators: A Systematic Review and Meta-Analysis of Randomized Controlled Trials and TSA

**DOI:** 10.3390/biomedicines14010207

**Published:** 2026-01-18

**Authors:** Liviu Ștefan Călin, Darie Ioan Andreescu, Mircea Ioan Alexandru Bistriceanu, Cosmin Gabriel Ursu, Andrei Constantin Anghel, Remus Valentin Anton, Vasile Bogdan Fodor, Maria Daria Răileanu, Cristian Valentin Toma, Gabriel Olteanu, Dragoș Alin Trache, Liviu Ionuț Șerbănoiu, Anamaria Georgiana Avram, Francesco Perone, Ștefan Sebastian Busnatu

**Affiliations:** 1 Bagdasar-Arseni Clinical Emergency Hospital, 041915 Bucharest, Romania; 2 Faculty of Medicine, Carol Davila University of Medicine and Pharmacy, 050474 Bucharest, Romania; 3 Department of Cardio-Thoracic Pathology, Faculty of Medicine, Carol Davila University of Medicine and Pharmacy, 050474 Bucharest, Romania; 4 Cardiac Rehabilitation Unit, Rehabilitation Clinic “Villa delle Magnolie”, Castel Morrone, 81020 Caserta, Italy

**Keywords:** cardiac rehabilitation, implantable cardioverter-defibrillator, peak VO_2_, exercise capacity

## Abstract

**Background/Objectives**: Cardiac rehabilitation (CR) is known to improve clinical outcomes in cardiovascular disease, yet its benefits in patients with implantable cardioverter-defibrillators (ICD) are not well established. This meta-analysis evaluated the impact of CR on functional capacity and safety in ICD recipients. **Methods**: A systematic search of PubMed, Scopus, and Cochrane Library was performed to identify randomized controlled trials (RCT) involving adults who underwent ICD implantation and were assigned to either CR or standard care. The primary outcome was the change in peak oxygen uptake (peak VO_2_) from the baseline to the final follow-up. Random-effects models were applied, and subgroup analyses were conducted based on follow-up duration, supervision type, baseline peak VO_2_, and ischemic vs. non-ischemic etiology. **Results**: Seven RCTs involving 1461 participants (784 CR; 677 control) met the inclusion criteria. CR was associated with a significant improvement peak VO_2_ compared with usual care, expressed as the mean difference (MD) in change from the baseline to the last follow-up (MD 2 mL·kg^−1^·min^−1^; 95% CI 1.02–2.81; I^2^ = 65.7%), with consistent effects across all subgroups. Quality of life improved in the CR group (MD 6.46; 95% CI 2.25–10.67; I^2^ = 0%). A non-significant trend toward increased 6MWT distance was observed. CR did not increase adverse events, including ICD shocks, hospitalizations, or cardiac deaths. **Conclusions**: CR safely enhances exercise capacity and quality of life in ICD recipients without increasing arrhythmic events or mortality. Larger standardized trials are warranted to optimize CR delivery in this population.

## 1. Introduction

Cardiac rehabilitation (CR) is a structured, multidisciplinary intervention that encompasses exercise training programs, patient education, and psychological support in order to improve prognosis and quality of life in individuals with cardiovascular disease [1]. Its benefits are well established in patients with coronary artery disease and chronic heart failure, including improved exercise capacity, reduced morbidity, and lower rates of hospital readmission [2,3].

Patients with implantable cardiac devices—such as implantable cardioverter-defibrillators (ICDs) and cardiac resynchronization therapy (CRT) systems—represent an expanding subgroup with distinctive characteristics in the context of CR. Historically, the possibility of device-related complications, including ICD shocks and lead dislocation, patients’ arrhythmogenic predisposition and the associated comorbidities of this subgroup, led to their limited referral and participation in CR programs [4]. Consequently, evidence that is specific to this population has remained relatively scarce compared to other cardiovascular cohorts.

The current state of the literature, however, suggests that CR is both safe and beneficial for patients with implantable devices. Reported advantages include improved functional capacity, reduced anxiety and depression, and lower hospitalization risk compared to standard care [5]. Despite these encouraging findings, uncertainty persists regarding the magnitude and durability of these benefits, the influence of individual clinical profiles, and the extent to which variability in CR interventions may shape patient outcomes in this population.

This meta-analysis aims to synthesize the current evidence and provide a comprehensive assessment of the efficacy and safety of CR compared with standard care in patients with ICDs, with a focus on refining outcome interpretation through stratified and exploratory analyses.

## 2. Materials and Methods

This systematic review and meta-analysis was conducted in accordance with the Preferred Reporting Items for Systematic Reviews and Meta-Analyses (PRISMA) guidelines (Supplementary Table S1) [6]. The review protocol was prospectively registered on PROSPERO (CRD420251163872).

### 2.1. Data Sources and Search Methods

We systematically searched PubMed, Scopus, and the Cochrane Central Register of Controlled Trials from inception to 20 September 2025, using the following search strategy: (ICD OR “cardiac devices” OR “defibrillator” OR “implantable cardioverter defibrillator”) AND (“cardiac rehabilitation” OR “telerehabilitation” OR “exercise training” OR “in-hospital rehabilitation” OR “cardiac exercise” OR “exercise-based cardiac rehabilitation” OR “physical therapy” OR “exercise training” OR “cardiac reconditioning” OR “physical rehabilitation” OR “physiotherapy” OR “exercise therapy” OR “exercise intervention” OR “multidisciplinary rehabilitation” OR “lifestyle intervention” OR “secondary prevention” OR “home-based rehabilitation”).

### 2.2. Study Eligibility

We included studies involving adult patients (≥18 years) who underwent CR after ICD implant, either during the initial hospital stay or follow-up. Eligible studies had to report comparative outcomes between the CR group and the standard of care group. Only ICD and CRT-D, and all clinical settings (elective, urgent, ischemic, or non-ischemic patients), were considered. Randomized controlled trials were only eligible if they provided original comparative data. Only articles published in English were included. Studies needed to report at least the primary outcome of interest, and peak VO_2_ change from baseline to last follow-up. We excluded studies of mixed populations (pacemakers or CRT-P) where ICD recipients could not be analyzed separately. Case reports, case series, editorials, narrative reviews, and studies without extractable data, as well as non-human or in vitro studies, were also excluded.

### 2.3. Selection of Studies

The references of all included studies, previous systematic reviews, and meta-analyses were also manually searched for additional studies. Randomized control trials that included patients after ICD implantation who underwent the CR program were eligible for this review. The initial screening was independently carried out by three authors (D.I.A., M.I.A.B., and L.S.C.) and retrieved if deemed potentially eligible by at least one reviewer. The same three reviewers independently evaluated full texts for eligibility. Any disagreements were addressed through discussion, and if a consensus could not be reached, a fourth reviewer (S.S.B.) served as an arbiter.

### 2.4. Data Extraction

Two reviewers (D.I.A. and M.I.A.B.) independently extracted data from the included studies. Study characteristics collected included authors, year of study, sample size and follow-up. Sample population characteristics included mean age (SD), female sex distribution, prevalence of hypertension, diabetes mellitus, atrial fibrillation, ischemic heart disease and ICD indication for primary prevention of sudden cardiac death. Data were independently extracted by two reviewers (D.I.A. and M.I.A.B.), using Microsoft Excel. If any critical data were missing or unclear, the corresponding authors of the studies were contacted via email, with a follow-up reminder sent after 1 week.

### 2.5. Assessment of Quality and Bias

The risk of bias was evaluated based on the study design. The Risk of Bias 2 (RoB 2) tool was utilized for all randomized controlled trials [7]. Two independent reviewers (D.I.A. and M.I.A.B.) carried out all risk of bias assessments. The other two reviewers (C.G.U and G.O.) independently rated the certainty of evidence using the Grading of Recommendations Assessment, Development, and Evaluation GRADEpro GDT (McMaster University, 2025) software. Certainty of evidence was graded as high, moderate, low, or very low and was downgraded if there were concerns with risk of bias, inconsistency, imprecision, or indirectness (Supplementary Table S2). Disagreements were resolved by discussion or by consultation with another author (S.S.B.).

### 2.6. Outcomes Consistency

The primary outcome is peak VO_2_ change (the difference value from the last follow-up to baseline). Secondary outcomes include peak VO_2_ at the baseline and at follow-up (less than 3 months, more than 3 months, last follow-up), peak VO_2_ at the last follow-up in directly supervised rehabilitation or remotely monitored, baseline peak VO_2_, QoL-SF-36 general health, 6MWT distance, ICD shocks, hospitalizations, and cardiac deaths during follow-up.

### 2.7. Data Synthesis and Analysis

Our meta-analysis used a therapeutic model. Binary outcomes were analyzed using odds ratios (ORs) with corresponding 95% confidence intervals (CIs). For continuous variables, treatment effects were estimated as mean differences (MDs) with 95% CIs. To decide which studies to include in each synthesis, we first compiled a table of each study’s characteristics, including population, intervention, comparator, outcome, and time point. We only included studies that provided sufficient data for the specific outcome of interest and matched the comparison groups in each meta-analysis. Two group comparisons of binary outcomes were conducted with the inverse variance model. Continuous outcomes were compared between patients who were included in the rehabilitation program and those that benefit from standard care. Given the heterogeneity in how CR was defined across studies, we prespecified and performed subgroup analyses for peak VO_2_ difference change, by follow-up—more or less than 3 months, type of rehabilitation (directly supervised/remotely monitored), only ICD or CRT-D, for baseline peak VO_2_—higher or less than 20 mL/kg/min, and in ischemic substrate predominance (studies that reported more or less than 60% of ischemic patients) to explore potential differences in outcomes.

A random-effects model was selected for this analysis to account for both within-study sampling errors and between-study heterogeneity. This method is recommended by Cochrane due to the variability in study designs, making it nearly impossible to have studies that are methodologically identical [8]. Between-study variance (τ^2^) was estimated using the restricted maximum-likelihood (REML) method, which remains a standard approach in random-effects meta-analyses due to its interpretability and reliable performance across different levels of heterogeneity. Heterogeneity was assessed using τ^2^, I^2^ (percentage of total variability attributable to heterogeneity), and H^2^ (ratio of total to within-study variability). Additionally, Cochran’s Q test was used to statistically evaluate the presence of heterogeneity. Because the Q test has limited power with smaller sample sizes, a significance level of 0.10 was used instead of the conventional 0.05, increasing the sensitivity to detect true heterogeneity. To assess the contribution of individual studies to the overall heterogeneity and their influence on the pooled effect size, the Baujat plot was generated using the meta package in R. The funnel plot was created to visually examine asymmetry in the distribution of effect sizes relative to their standard errors. Subsequently, the asymmetry test (Egger’s test) was employed to evaluate publication bias quantitatively.

Subsequently, in order to evaluate the risk of random errors and determine whether the cumulative evidence was adequately powered, we performed a trial sequential analysis (TSA) using the RTSA package in R. The analysis was conducted for a continuous outcome, expressed as a mean difference. A two-sided type I error of 5% and type II error of 20% (power 80%) were prespecified. O’Brien–Fleming-type alpha and beta spending functions were applied for the construction of sequential monitoring boundaries. A non-binding futility boundary was used. For TSA, the required information size (RIS) was estimated under a diversity-adjusted random-effects model, using the DerSimonian–Laird estimator with the Hartung–Knapp–Sidik–Jonkman correction. Cumulative Z-curves were plotted against the sequential monitoring boundaries to assess whether firm evidence had been reached or whether further studies would be required.

R version 4.5.0 (R Foundation for Statistical Computing, Vienna, Austria) was utilized for all statistical analyses.

## 3. Results

### 3.1. Study Characteristics

**Figure 1:**
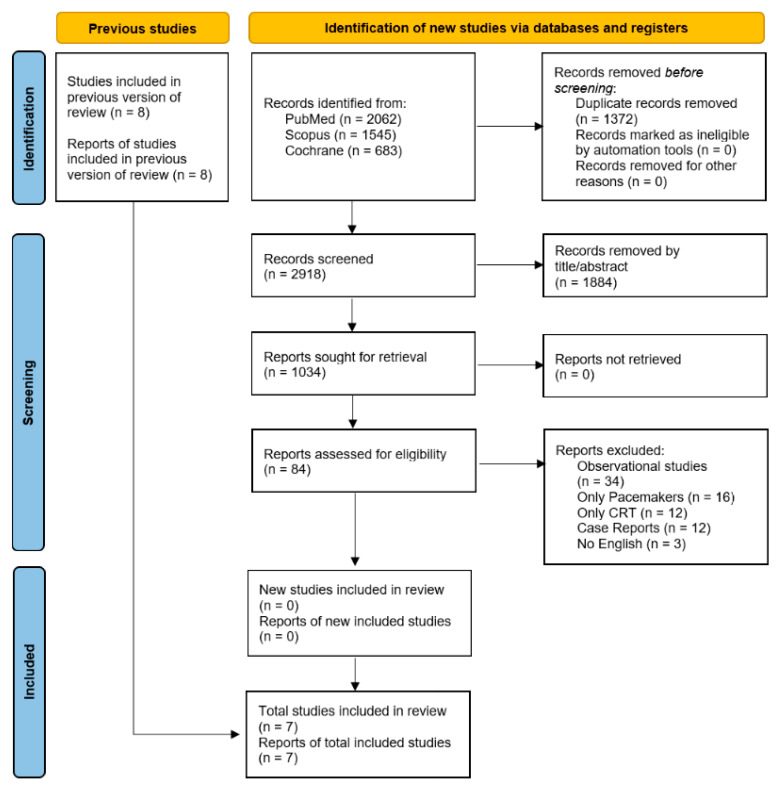
Preferred Reporting Items for Systematic Reviews and Meta-Analyses (PRISMA) flow diagram.

The systematic search yielded 4290 records, of which 1034 unique articles were screened. After full-text evaluation, seven randomized controlled trials fulfilled the eligibility criteria and were included in the meta-analysis (Figure 1) [9,10,11,12,13,14,15]. In total, 1461 patients were analyzed, including 784 in the CR group and 677 in the control group. The baseline characteristics were comparable between groups, with a mean age of 61.8 ± 12.91 years in the CR group and 61.12 ± 12.05 years in the standard of care group. The proportion of female patients was similar (18.8% vs. 19.8%). The weighted median follow-up across the included studies was 3 months (range 2–18 months). Other baseline patient characteristics are presented in Table 1.

### 3.2. Outcome Analysis

**Figure 2:**
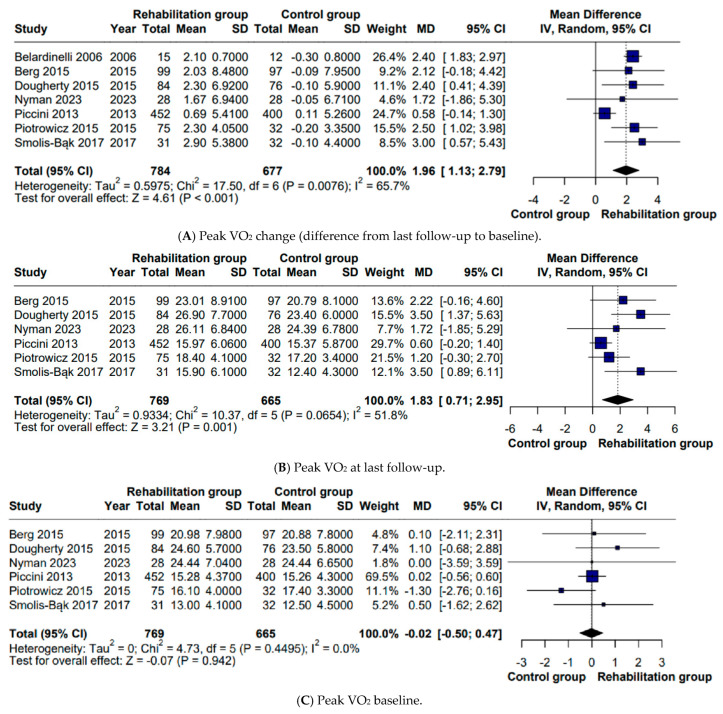
Forest plots of pooled analyses of peak VO_2_: (**A**) change from baseline to last follow-up; (**B**) at last follow-up; (**C**) at baseline [9,10,11,12,13,14,15]. Squares represent individual study effect estimates, with sizes proportional to study weight; horizontal lines indicate 95% confidence intervals, and the diamond represents the pooled effect estimate. The vertical line represents the line of no effect (MD = 0).

Across seven studies including 784 rehabilitation and 677 control patients, participation in CR was associated with a significant increase in peak VO_2_ from the baseline to the end of follow-up (MD 1.9 mL·kg^−1^·min^−1^, 95% CI 1.13–2.79; I^2^ = 65.7%), and this benefit remained evident when analyzing peak VO_2_ at the final assessment (MD 1.83 mL·kg^−1^·min^−1^, 95% CI 0.71–2.95; I^2^ = 51.8%). No differences were present at the baseline, confirming a comparable starting capacity (Figure 2).

**Figure 3:**
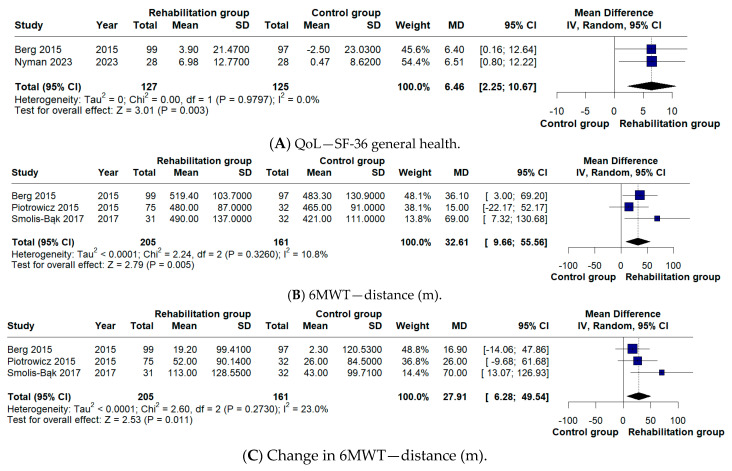
(**A**,**B**) Forest plots of health-related quality of life (SF-36 General Health score) and 6-min walk test (6MWT) distance assessed at follow-up; (**C**) forest plot of the change in 6MWT distance from baseline to follow-up. [9,10,11,13]. Squares represent individual study effect estimates, with sizes proportional to study weight; horizontal lines indicate 95% confidence intervals, and the diamond represents the pooled effect estimate. The vertical line represents the line of no effect (MD = 0).

CR also led to a significant improvement in health-related quality of life, with higher SF-36 General Health scores compared with controls (MD 6.46, 95% CI 2.25–10.67; I^2^ = 0%). Although changes in 6-min walk distance favored the intervention, the pooled estimates did not reach statistical significance (Figure 3).

**Figure 4:**
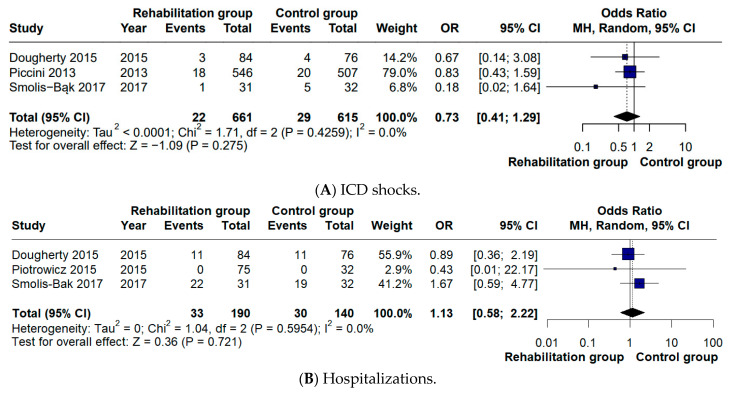

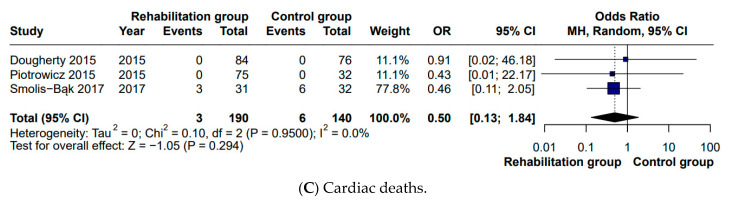
Forest plots of clinical outcomes during follow-up: (**A**) ICD shocks; (**B**) hospitalizations; (**C**) cardiac deaths [10,11,12,13,14]. Squares represent individual study effect estimates, with sizes proportional to study weight; horizontal lines indicate 95% confidence intervals, and the diamond represents the pooled effect estimate. The vertical line represents the line of no effect (OR = 1).

No group differences were observed for appropriate or inappropriate ICD shocks (OR 0.73, 95% CI 0.41–1.29), all-cause hospitalizations (OR 1.13, 95% CI 0.58–2.22), or cardiac mortality (OR 0.50, 95% CI 0.13–1.84) (Figure 4).

### 3.3. Subgroup Analyses

Subgroup analyses showed consistent improvements in peak VO_2_ across follow-up durations, with significant effects in both short-term (MD 1.80, 95% CI 0.81–2.79; I^2^ = 75.3%) and longer-term studies (MD 2.64, 95% CI 1.10–4.18; I^2^ = 0%) (see Supplementary Material online, Figure S1). The benefits were also evident across supervised programs (MD 1.80, 95% CI 0.73–2.88) and remote interventions (MD 2.46, 95% CI 1.28–3.65) (see Supplementary Material online, Figure S2) and were particularly strong in ICD-only cohorts (MD 2.41, 95% CI 1.89–2.94; I^2^ = 0%) (see Supplementary Material online, Figure S3). Similar improvements were observed regardless of baseline functional capacity (see Supplementary Material online, Figure S4) or ischemic etiology (see Supplementary Material online, Figure S5), indicating that the positive effect of CR on exercise capacity was broad and consistent across patient subgroups.

### 3.4. Publication Bias

The funnel plot demonstrated a symmetrical distribution of studies, and Egger’s test did not provide evidence of small-study effects (intercept 0.52, 95% CI −1.93 to 2.97; *p* = 0.69), indicating no significant publication bias (Figure S6). The Baujat plot identified Piccini as contributing the most to heterogeneity and exerting the greatest influence on the pooled effect, while all other studies showed minimal impact (see Supplementary Material online, Figure S7). However, even with Piccini included, the overall direction and significance of the pooled effect remained unchanged, suggesting that no single study disproportionately distorted the main findings.

### 3.5. Risk of Bias Assessment

The methodological quality of the randomized controlled trials included was evaluated using the RoB 2 tool. Most trials demonstrated adequate randomization procedures and low risk in outcome measurement and the selection of the reported result domains (see Supplementary Material online, Figure S8). One study (Piotrowicz [13]) was evaluated as high risk as a result of the baseline imbalances between groups that were observed after the randomization process. However, concerns were identified primarily in the domains of missing outcome data, which are often related to incomplete reporting of adherence or high attrition rates. As a consequence, two additional studies (Smolis-Bąk [10] and Piccini [14]) were rated as a high overall risk. Given the nature of rehabilitation programs, intervention blinding was not achievable; however, it was not considered a factor that could significantly impact results. Despite these limitations, the overall quality of the evidence was considered acceptable, with no systematic bias expected to significantly alter the main findings.

### 3.6. Trial Sequential Analysis

**Figure 5:**
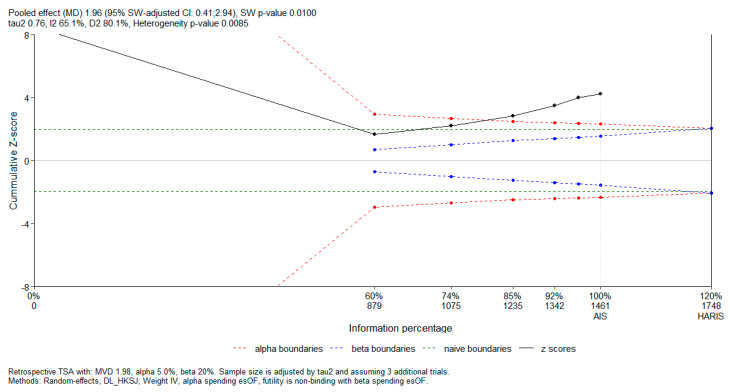
Trial sequential analysis of primary outcome.

Previously, a very thorough meta-analysis performed by Nielsen et al. [16] published in the Cochrane Database of Systematic Reviews showed that there is indeed a statistically significant difference. To that end, we performed a TSA for our primary outcome, the peak VO_2_ difference between the baseline and the last follow-up, to verify whether the total sample size has become large enough to overcome heterogeneity and uncertainty. Our heterogeneity-adjusted required information size (HARIS) is 1748 patients, which is only 287 patients away from the current sample size, while confidently crossing the alpha boundary without entering the futility zone. This shows that while the results are statistically significant and look very promising, there is still a need for further studies to have certainty on the effect of rehabilitation. The TSA plot showing our results is included in Figure 5.

## 4. Discussion

We performed an updated meta-analysis to focus specifically on patients with implantable cardioverter-defibrillators undergoing CR, with functional capacity—particularly peak VO_2_—as a primary outcome. While a previous meta-analysis published in 2019 [16] also examined ICD recipients, its main focus was mortality, and peak VO_2_ was addressed only as a secondary endpoint. In contrast, our study provides a more detailed and up-to-date assessment of exercise capacity in this distinct population. Most of the evidence exploring exercise capacity in device recipients has come from broader meta-analyses that combined patients with different implantable devices, including ICDs, CRT devices, and pacemakers [5], or from studies that were restricted solely to CRT recipients [17,18]. By isolating patients with ICDs, our analysis provides a more detailed evaluation of this specific category, thereby addressing an important gap in the literature. The primary outcome was peak oxygen consumption, a well established marker of exercise capacity and a strong prognostic indicator in patients with cardiovascular disease. Similarly to Kaddoura et al. [5], who evaluated a subgroup containing ICD patients (MD 2.08 mL/kg/min), our findings confirm that CR is associated with a significant improvement in VO_2_ peak among ICD recipients (MD 2 mL/kg/min). Our work offers an additional level of insight by performing multiple subgroup analyses, which allowed for a more nuanced understanding of factors that may influence the exercise response. Statistically significant differences were observed regardless of etiology—predominantly ischemic (MD 1.91 mL/kg/min) or mixed (MD 2.28 mL/kg/min), baseline peak VO_2_—>20 mL/kg/min, corresponding to Weber class A (MD 2.20 mL/kg/min) or ≤20 mL/kg/min, including Weber classes B and C (MD 1.93 mL/kg/min), and supervision modality—and directly supervised (MD 1.80 mL/kg/min) or remotely monitored (MD 2.46 mL/kg/min). These subgroup observations are particularly relevant in light of the broader shift toward personalized interventions. While improvements in peak VO_2_ following CR have been reported consistently across studies, other aspects of exercise prescription and patient selection remain less clearly defined. This underscores the need to explore which conditions patients should exercise under, which training modalities are the most appropriate, and how to better identify candidates based on parameters such as baseline peak VO_2_ and underlying etiology.

The studies were also stratified according to follow-up duration, distinguishing between interventions of ≤3 months and those exceeding 3 months. A statistically significant difference was observed only in the subgroup with follow-up periods of 3 months or less (MD 1.80 mL/kg/min). This finding suggests that the benefits of CR may become apparent relatively early. However, the absence of a statistically significant effect in the subgroup with longer follow-up should not be interpreted as evidence of inefficacy or insufficient intervention duration. Rather, it is more plausibly explained by the very limited number of studies available for this comparison, as only two trials were included in this subgroup. The primary outcome was also examined in relation to device status. Studies that included only ICD recipients were compared with those in which a proportion of patients had CRT devices. A significant improvement in peak VO_2_ was only found in the ICD-only subgroup (MD 2.41 mL/kg/min), whereas mixed ICD/CRT samples did not show a comparable effect (MD 1.43 mL/kg/min). This pattern may suggest that the clinical profile of patients who require CRT—characterized by more advanced cardiac dysfunction and a higher burden of comorbidities [19,20]—could attenuate the response to rehabilitation.

The heterogeneity observed in peak VO_2_ outcomes may also be partly explained by differences in the cardiac rehabilitation protocols applied across the included RCTs. Nyman et al. [9] implemented a supervised high-intensity interval training program consisting of four 4 min intervals at 85–95% of the maximum heart rate, performed three times weekly for 12 weeks. In contrast, Belardinelli et al. [15] and Berg et al. [11] employed supervised, center-based moderate-intensity continuous aerobic exercise programs, with training intensity generally maintained at submaximal levels. Dougherty et al. [12] evaluated a structured home-based aerobic rehabilitation program with predefined exercise prescriptions and periodic supervision, while Piotrowicz et al. [13] and Smolis-Bąk et al. [10] assessed telemonitored or remotely supervised CR models, emphasizing safety and adherence in ICD recipients. Piccini et al. [14] focused on moderate-intensity exercise training, combined with structured follow-up and quality-of-life assessment. Such protocol-related differences—particularly regarding exercise intensity, supervision, and delivery modality—may contribute to variability in effect size estimates and partially account for the moderate heterogeneity observed in pooled analyses. Higher-intensity and fully supervised programs are generally associated with larger improvements in cardiorespiratory fitness, whereas home-based or remotely supervised interventions may yield more modest gains. Nevertheless, despite these variations, all included interventions represented structured aerobic rehabilitation programs targeting similar physiological endpoints. Importantly, the direction of effect consistently favored CR across studies, suggesting that protocol heterogeneity is more likely to influence the magnitude of, rather than the presence of, the benefit.

Importantly, the observed increase of approximately 2 mL/kg/min in peak VO_2_ is clinically meaningful. Peak VO_2_ is a strong integrative marker of cardiovascular, pulmonary, and muscular function, and even modest improvements have been consistently associated with better functional status and prognosis in patients with cardiovascular disease. Contemporary evidence indicates that improvements in exercise capacity achieved through CR translate into clinically relevant gains, including improved functional class and long-term outcomes [1]. In this context, the magnitude of benefit observed in the present meta-analysis is likely to be of clinical relevance for ICD recipients.

In terms of secondary outcomes, our analysis explored changes in 6MWT distance, cardiac mortality, hospitalization rates, incidence of ICD shocks, and quality of life. With respect to 6MWT distance, our meta-analysis did not identify a significant difference (MD 27.91 m), unlike the findings reported by Kaddoura et al. [5] (MD 41.51 m). This is not due to differences in study inclusion, as the same trials were analyzed. Instead, the discrepancy arises from the handling of data in Berg et al. [11]. In our analysis, the 6-month follow-up values were compared with those at 3 months, which reflect the pre-rehabilitation assessment, whereas Kaddoura et al. [5] compared the 6-month values with the baseline measurements recorded at randomization.

While the meta-analysis by Kaddoura et al. [5] reported no difference in all-cause mortality, our analysis specifically examined cardiac mortality and likewise found no significant difference between groups (OR 0.46). In studies where all-cause mortality was reported as zero, we inferred cardiac mortality to be absent as well [12,13]. Consistent with the findings reported in the previous meta-analysis, no significant differences were detected in hospitalization rates (OR 1.16) or in the number of ICD shocks (OR 0.73). With respect to quality of life, a positive effect in favor of CR was identified in our analysis (MD 6.46), whereas this outcome had not been statistically assessed in the previous meta-analysis [5]. These outcomes are particularly relevant since evidence from recent studies indicates that ICD shocks can negatively influence both objectively recorded physical activity and patients’ quality of life. Moreover, pooled analyses of large ICD cohorts suggest an association between shock occurrence—especially appropriate shocks for rapid ventricular arrhythmias—and increased mortality risk [21,22]. These neutral findings should be interpreted with caution, as the number of available trials and clinical events for these secondary outcomes was limited, resulting in reduced statistical power to detect significant differences. Overall, these results update and refine the current evidence on CR in ICD recipients, clarifying its impact on functional capacity while contextualizing secondary clinical outcomes.

### 4.1. Strengths

This meta-analysis has several methodological and analytical strengths. Restricting the evidence base to RCTs increases methodological rigor and minimizes bias compared to observational or mixed designs. A key distinction from previous meta-analyses is that the present work aimed to direct focus strictly on patients with ICDs, which enhances the specificity and clinical relevance of the findings. In contrast, the meta-analysis by Kaddoura et al. (2024) [5] included a broader population with various implantable cardiac devices (ICDs, CRTs, and pacemakers). Although that study also analyzed ICD patients separately, our work exceeds the ICD patients pool by incorporating additional studies. Another meta-analysis by Nielsen et al. (2019) [16] implemented a different design by adopting all-cause mortality as a primary outcome, rather than peak VO2 change, which resulted in a partially different selection of articles. It is noteworthy that peak VO_2_ was analyzed as a post-intervention absolute value, rather than as a change from the baseline. A major advantage is the depth of the subgroup analyses. The intervention setting was examined by differentiating between directly supervised [9,10,11,14,15] and remotely monitored [12,13] exercise programs. While remotely monitored programs were delivered exclusively in the home setting, supervised interventions took place in center-based [10,14,15], home-based, or mixed formats [11]. The patient phenotype was also accounted for by stratifying outcomes according to the baseline peak VO_2_ and heart failure etiology. For baseline functional capacity, the analysis compared Weber class A (peak VO_2_ > 20 mL/kg/min) versus classes B–C (<20 mL/kg/min). Etiology was classified as predominantly ischemic when >60% of the sample had ischemic heart disease [9,10,13,14,15], and as mixed when the proportion ranged between 40 and 60% [11,12]. None of the included studies reported an ischemic etiology below 40%. The length of follow-up was also examined by means of subgroup stratification, distinguishing between short-term outcomes (≤3 months) and longer-term outcomes (>3 months). Although previous meta-analyses addressed all-cause mortality, the present work provides a statistical pooled estimate of cardiac mortality. Likewise, quality of life was quantitatively synthesized using the Short Form-36 questionnaire, an outcome that was mentioned descriptively—but not pooled—in the analyses by Kaddoura et al. [5] and Nielsen et al. [16].

### 4.2. Limitations

Despite its strengths, this meta-analysis also presents important limitations. Regarding risk of bias, three included studies presented high overall risk—one of them [13] particularly, due to baseline imbalances between groups (e.g., age, left ventricular ejection fraction, heart failure etiology)—while in the other two trials, there was loss to follow-up, which was likely related to clinical deterioration or death [10,14]. In addition, one of the included studies was only available as an abstract (Nyman et al. [9]), which limited access to detailed information on randomization procedures, outcome assessment, and protocol adherence. This may introduce additional uncertainty regarding methodological quality and effect size estimation, although the study met the predefined inclusion criteria and contributed a limited weight to the pooled analyses. Dropout rates reached approximately 25% in the studies by Smolis-Bąk et al. [10] and Berg et al. [11], and around 20% in Piccini et al. [14]. Another methodological limitation is that, in three of the seven included studies, patients with ICDs were analyzed together with those receiving CRT, and separate outcome data for ICD patients could not be extracted [9,13,14]. The proportion of patients with CRT was 32.71% in Piotrowicz et al.’s study [13], 41.31% in Piccini et al.’s study [14], and 46.42% in Nyman et al.’s study [9]. Although these patients were not the majority, their inclusion introduces heterogeneity in the underlying cardiac substrate and response to exercise.

The number of eligible RCTs was relatively small, especially within subgroup analyses, which limits both statistical power and the robustness of analyses exploring treatment effect modifiers. An additional limitation is that the persistence of the rehabilitation effect could not be evaluated, because the timing of the follow-up assessments of peak VO_2_ was not consistent across studies. For example, Dougherty et al. [12] assessed peak VO_2_ at 8 and 24 weeks, while Smolis-Bąk et al. [10] reported values at 6 and 18 months, preventing the construction of pooled longitudinal trajectories. The evaluation of quality of life was limited by variability in the assessment tools. Although SF-36 was the most frequently used instrument, Piotrowicz [13] employed a Polish adaptation of it, whereas Belardinelli [15] used the Minnesota Living with Heart Failure Questionnaire, and these instruments cannot be interconverted. Regarding left ventricular ejection fraction, this parameter was reported in only one study (Smolis-Bąk et al. [10]) and was not available for the ICD subgroup in Belardinelli et al. [15], which precluded its inclusion as an outcome reflecting potential cardiac functional improvement.

Finally, exercise prescription was not standardized across the included trials. Considerable variability existed in training mode, intensity, supervision, and duration, which may contribute to heterogeneity in outcomes and limit the direct translation of these findings into uniform clinical practice.

## 5. Conclusions

This updated meta-analysis demonstrates that CR significantly improves functional capacity in patients with implantable cardioverter-defibrillators, as reflected by the increased peak VO_2_. The benefit was consistent across subgroups defined by baseline fitness, etiology, duration of follow-up, and rehabilitation setting, highlighting the robustness and generalizability of the effect. Quality of life also improved, while no differences were observed in ICD shocks, hospitalization rates, or cardiac mortality, confirming the safety of rehabilitation in this population. Together, these findings reinforce the role of exercise-based CR as a safe and effective intervention for ICD recipients, providing both physiological and psychosocial benefits. Future large-scale, standardized randomized controlled trials are warranted to optimize the program structure, clarify the long-term effects, and define the patient phenotypes that may derive the greatest benefit.

## Figures and Tables

**Table 1:** Baseline characteristics of patients.

Study	Year	Sample Size	Age (Years)	Female n (%)	Hypertension n (%)	Atrial Fibrillation n (%)	Diabetes n (%)	Ischemic Heart Disease n (%)	ICD in Primary Prevention n (%)	Follow-Up (Months)
Nyman [9]	2023	56	68.3 ± 9.7/68.1 ± 7.3	4 (14)/6 (21)	12 (43)/ 11 (39)	15 (54)/ 3 (11)	9 (32)/4 (14)	37 (66.1%)	23 (41%)	3
Smolis-Bąk [10]	2017	84	63.7 ± 9.5/61.1 ± 9.7	5 (12.1)/3 (6.9)	27 (65.9)/ 24 (55.8)	14 (34.1)/23 (53.4)	13 (31.7)/15 (34.9)	49 (77.7%)	84 (100%)	18
Berg [11]	2015	196	57.6 ± 12.9/56.7 ± 13.5	20 (20.2)/21 (21.6)	18 (18)/ 23 (24)	27 (27)/ 21 (22)	12 (12)/10 (10)	102 (52%)	130 (66.3%)	3
Dougherty [12]	2015	160	56.1 ± 12.1/53.6 ± 12.2	17 (20.2)/19 (25.0)	NA	NA	NA	69 (43.1%)	68 (42.5%)	6
Piotrowicz [13]	2015	107	54.4 ± 10.9/62.1 ± 12.5	11 (15)/1 (3)	NA	NA	15 (20.0)/7 (21.9)	77 (72%)	NA	2
Piccini [14]	2013	852	64.4 ± 12.6/63 ± 11.1	113 (21)/109 (21)	314 (58)/ 274 (54)	155 (28)/140 (28)	183 (34)/167 (33)	644 (61.5%)	NA	3
Belardinelli [15]	2006	27	55.1 ± 14/53.1 ± 15	0 (0)/0 (0)	NA	NA	NA	27 (100%)	NA	2

ICD = implantable cardioverter defibrillator, NA = not available (NA indicates that the respective data were not reported in the original trial publications and could therefore not be extracted); age is described as mean plus standard deviation.

## Data Availability

This study is based on previously published data. No new data were generated or analyzed.

## Supplementary Materials

The following supporting information can be downloaded at: https://www.mdpi.com/article/10.3390/biomedicines14010207/s1, Figure S1. Forest plots of peak VO_2_ change at less or more than 3 months follow-up; Figure S2. Forest plots of peak VO_2_ change in directly supervised or remotely monitored cardiac rehabilitation; Figure S3. Forest plots of subgroups analysis regarding peak VO_2_ change in only ICD patients or mixed populations; Figure S4. Forest plots of subgroups analysis regarding baseline peak VO_2_; Figure S5. Forest plots of subgroup analysis regarding baseline peak VO_2_ and ischemic etiology; Figure S6. Publication Bias and Eggers’ test; Figure S7. Baujat plot; Figure S8. Risk of bias graph and summary; Table S1. PRISMA checklist; Table S2. Certainty of evidence— GRADEpro GDT assessment.

## Abbreviations

The following abbreviations are used in this manuscript:

CI	confidence interval
CR	cardiac rehabilitation
CRT	cardiac resynchronization therapy
ICD	implantable cardioverter-defibrillators
MD	mean difference
6MWT	6-min walk test
NA	not available
OR	odds ratio
Peak VO_2_	peak oxygen uptake
PRISMA	The Preferred Reporting Items for Systematic Reviews and Meta-Analyses
RCT	randomized controlled trials
REML	restricted maximum-likelihood
RoB2	The Risk of Bias 2
SD	standard deviation
