# Supplementary material for: Cardiac Rehabilitation in Patients with Implantable Cardioverter-Defibrillators: A Systematic Review and Meta-Analysis of Randomized Controlled Trials and TSA
Source: Biomedicines. 2026 Jan 18;14(1):207. doi: 10.3390/biomedicines14010207 (PMC12839275; doi:10.3390/biomedicines14010207)
Supplement: Supplementary file 1 [file biomedicines-14-00207-s001.zip › biomedicines-4094201-supplementary.pdf]

Section and Topic	Item #	Checklist item	Location where item is reported
**TITLE**			
Title	1	Identify the report as a systematic review.	Pg. 1, Lines 2-3
**ABSTRACT**			
Abstract	2	See the PRISMA 2020 for Abstracts checklist.	Pg. 1-2, Lines 27-44
**INTRODUCTION**			
Rationale	3	Describe the rationale for the review in the context of existing knowledge.	Pg. 2, Lines 60-64
Objectives	4	Provide an explicit statement of the objective(s) or question(s) the review addresses.	Pg. 2, Lines 65-67
**METHODS**			
Eligibility criteria	5	Specify the inclusion and exclusion criteria for the review and how studies were grouped for the syntheses.	Pg. 3, Lines 82-88
Information sources	6	Specify all databases, registers, websites, organisations, reference lists and other sources searched or consulted to identify studies. Specify the date when each source was last searched or consulted.	Pg.3, Lines 74-79
Search strategy	7	Present the full search strategies for all databases, registers and websites, including any filters and limits used.	Pg.3, Lines 74-79
Selection process	8	Specify the methods used to decide whether a study met the inclusion criteria of the review, including how many reviewers screened each record and each report retrieved, whether they worked independently, and if applicable, details of automation tools used in the process.	Pg. 3, Lines 90-95
Data collection process	9	Specify the methods used to collect data from reports, including how many reviewers collected data from each report, whether they worked independently, any processes for obtaining or confirming data from study investigators, and if applicable, details of automation tools used in the process.	Pg.3, Lines 97-103
Data items	10a	List and define all outcomes for which data were sought. Specify whether all results that were compatible with each outcome domain in each study were sought (e.g. for all measures, time points, analyses), and if not, the methods used to decide which results to collect.	Pg. 4, Lines 112-116
	10b	List and define all other variables for which data were sought (e.g. participant and intervention characteristics, funding sources). Describe any assumptions made about any missing or unclear information.	Pg.3, Lines 101-103
Study risk of bias assessment	11	Specify the methods used to assess risk of bias in the included studies, including details of the tool(s) used, how many reviewers assessed each study and whether they worked independently, and if applicable, details of automation tools used in the process.	Pg.3, Lines 106-107
Effect measures	12	Specify for each outcome the effect measure(s) (e.g. risk ratio, mean difference) used in the synthesis or presentation of results.	Pg.4, Lines 119- 125
Synthesis methods	13a	Describe the processes used to decide which studies were eligible for each synthesis (e.g. tabulating the study intervention characteristics and comparing against the planned groups for each synthesis (item #5)).	Pg.4, Lines 118-151
	13b	Describe any methods required to prepare the data for presentation or synthesis, such as handling of missing summary statistics, or data conversions.	Pg.4, Lines 118-151

Section and Topic	Item #	Checklist item	Location where item is reported
	13c	Describe any methods used to tabulate or visually display results of individual studies and syntheses.	Pg.4, Lines 118-151
	13d	Describe any methods used to synthesize results and provide a rationale for the choice(s). If meta-analysis was performed, describe the model(s), method(s) to identify the presence and extent of statistical heterogeneity, and software package(s) used.	Pg.4, Lines 130-140
	13e	Describe any methods used to explore possible causes of heterogeneity among study results (e.g. subgroup analysis, meta-regression).	Pg.4, Lines 125-128
	13f	Describe any sensitivity analyses conducted to assess robustness of the synthesized results.	Pg.4, Lines 118-151
Reporting bias assessment	14	Describe any methods used to assess risk of bias due to missing results in a synthesis (arising from reporting biases).	Pg.4, Lines 118-151
Certainty assessment	15	Describe any methods used to assess certainty (or confidence) in the body of evidence for an outcome.	Pg.3, Lines 107-111
**RESULTS**			
Study selection	16a	Describe the results of the search and selection process, from the number of records identified in the search to the number of studies included in the review, ideally using a flow diagram.	Pg.5, Lines 160-161
	16b	Cite studies that might appear to meet the inclusion criteria, but which were excluded, and explain why they were excluded.	Pg.5, Figure 1
Study characteristics	17	Cite each included study and present its characteristics.	Pg. 6, Table 1
Risk of bias in studies	18	Present assessments of risk of bias for each included study.	Pg.10, Lines 275-283
Results of individual studies	19	For all outcomes, present, for each study: (a) summary statistics for each group (where appropriate) and (b) an effect estimate and its precision (e.g. confidence/credible interval), ideally using structured tables or plots.	Pg.6-9, Lines 171-256
Results of syntheses	20a	For each synthesis, briefly summarise the characteristics and risk of bias among contributing studies.	Pg.6-10
	20b	Present results of all statistical syntheses conducted. If meta-analysis was done, present for each the summary estimate and its precision (e.g. confidence/credible interval) and measures of statistical heterogeneity. If comparing groups, describe the direction of the effect.	Pg.6-10
	20c	Present results of all investigations of possible causes of heterogeneity among study results.	Pg. 9, Lines 268-273
	20d	Present results of all sensitivity analyses conducted to assess the robustness of the synthesized results.	Pg.9, Lines 257-265
Reporting biases	21	Present assessments of risk of bias due to missing results (arising from reporting biases) for each synthesis assessed.	Not applicable
Certainty of evidence	22	Present assessments of certainty (or confidence) in the body of evidence for each outcome assessed.	Supplementary material
**DISCUSSION**			
Discussion	23a	Provide a general interpretation of the results in the context of other evidence.	Pg.11-12,

Section and Topic	Item #	Checklist item	Location where item is reported
			Lines 299-347
	23b	Discuss any limitations of the evidence included in the review.	Pg.12, Lines 370-394
	23c	Discuss any limitations of the review processes used.	Pg.12, Lines 370-394
	23d	Discuss implications of the results for practice, policy, and future research.	Pg.12, Lines 350-369
**OTHER INFORMATION**			
Registration and protocol	24a	Provide registration information for the review, including register name and registration number, or state that the review was not registered.	Pg.3, Lines 69-71
	24b	Indicate where the review protocol can be accessed, or state that a protocol was not prepared.	Pg.3, Lines 69-71
	24c	Describe and explain any amendments to information provided at registration or in the protocol.	Pg.3, Lines 69-71
Support	25	Describe sources of financial or non-financial support for the review, and the role of the funders or sponsors in the review.	Pg. 14, Lines 422, 428-429
Competing interests	26	Declare any competing interests of review authors.	Pg.14, Line 431
Availability of data, code and other materials	27	Report which of the following are publicly available and where they can be found: template data collection forms; data extracted from included studies; data used for all analyses; analytic code; any other materials used in the review.	Pg.13, Lines 404-413

Table S1. PRISMA checklist

Cardiac rehabilitation compared to Standard of care for ICD pacients

Patient or population: ICD pacients

Intervention: Cardiac rehabilitation

Comparison: Standard of care

Outcomes	№ of participants (studies) Follow-up	Certainty of the evidence (GRADE)	Relative effect (95% CI)	Anticipated absolute effects	
				Risk with Standard of care	Risk difference with Cardiac rehabilitation
Peak VO2 change assessed with: mL·kg^{-1}·min^{-1} follow-up: range 2 months to 18 months	1461 (7 RCTs)	⨁⨁⨁◯ Moderate[a]	-		MD **1.96 higher** (1.13 higher to 2.79 higher)
Peak VO2 at last follow-up assessed with: mL·kg^{-1}·min^{-1}	1434 (6 RCTs)	⨁⨁⨁◯ Moderate[a]	-		MD **1.83 mL·kg^{-1}·min^{-1} higher** (0.71 higher to 2.95 higher)
Peak VO2 at baseline assessed with: mL·kg^{-1}·min^{-1}	1434 (6 RCTs)	⨁⨁⨁⨁ High	-		MD **0.02 mL·kg^{-1}·min^{-1} lower** (0.5 lower to 0.47 higher)
QoL-SF-36 general health	252 (2 RCTs)	⨁◯◯◯ Very low[b,c,d,e]	-		MD **6.46 higher** (2.25 higher to 10.67 higher)
Change in 6MWT - distance (m)	366 (3 RCTs)	⨁◯◯◯ Very low[f,g,h]	-		MD **27.91 m higher** (6.28 higher to 49.54 higher)

***The risk in the intervention group** (and its 95% confidence interval) is based on the assumed risk in the comparison group and the **relative effect** of the intervention (and its 95% CI).

CI: confidence interval; **MD:** mean difference

Cardiac rehabilitation compared to Standard of care for ICD pacients

Patient or population: ICD pacients

Intervention: Cardiac rehabilitation

Comparison: Standard of care

Outcomes	№ of participants (studies) Follow-up	Certainty of the evidence (GRADE)	Relative effect (95% CI)	Anticipated absolute effects	
				Risk with Standard of care	Risk difference with Cardiac rehabilitation

GRADE Working Group grades of evidence
High certainty: we are very confident that the true effect lies close to that of the estimate of the effect.
Moderate certainty: we are moderately confident in the effect estimate: the true effect is likely to be close to the estimate of the effect, but there is a possibility that it is substantially different.
Low certainty: our confidence in the effect estimate is limited: the true effect may be substantially different from the estimate of the effect.
Very low certainty: we have very little confidence in the effect estimate: the true effect is likely to be substantially different from the estimate of effect.

Explanations

a. Seven RCTs; three had overall high RoB (baseline imbalances in Piotrowicz, high attrition and incomplete outcome data in Smolis-Bąk and Piccini; one study available only as an abstract with limited methodological detail). Blinding of participants not feasible but outcome (peak VO_2) objective. Overall, some concerns that bias may influence effect estimate, so downgraded one level.

b. Both trials have limitations: no blinding of participants, subjective self-reported outcome (QoL), missing data and selective reporting risks. Overall high risk of bias for this patient-reported measure.

c. Population, intervention, comparator, and outcome directly address the research question. Use of validated SF-36 general health subscale is appropriate.

d. Only two studies available with notable differences in magnitude of benefit; the confidence intervals are wide and suggest important heterogeneity. Insufficient studies to explore heterogeneity.

e. Total sample size is small (<400), CI is wide (difference may be small or large), and optimal information size not met. Downgraded for imprecision.

f. Trials contributing to 6MWT have limitations: lack of blinding, incomplete outcome data, selective reporting concerns, and subjective patient effort inherent to the 6MWT. Overall, risk of bias is high or unclear in several key domains.

g. Effect estimates vary substantially among the trials, with wide and non-overlapping confidence intervals. Heterogeneity is high and cannot be explained with only 3 studies.

h. Total sample size is small; optimal information size is not met. The confidence interval includes both a small benefit (~6 m) and a clinically meaningful benefit (~50 m).

Table S2. Certainty of evidence— GRADEpro GDT assessment.

A. FOLLOW-UP > 3 months

Study	Year	Rehabilitation group Total	Mean	SD	Control group Total	Mean	SD	Weight	MD	95% CI
Dougherty 2015	2015	84	2.30	6.9200	76	-0.10	5.9000	59.9%	2.40	[0.41; 4.39]
Smolis-Bąk 2017	2017	31	2.90	5.3800	32	-0.10	4.4000	40.1%	3.00	[0.57; 5.43]
**Total (95% CI)**		**115**			**108**			**100.0%**	**2.64**	**[1.10; 4.18]**

Heterogeneity: $Tau^2 = 0$; $Chi^2 = 0.14$, df = 1 (P = 0.7080); $I^2 = 0.0\%$
Test for overall effect: Z = 3.36 (P < 0.001)

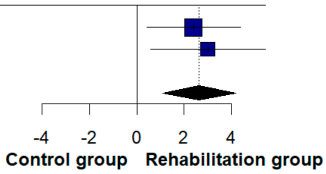

B. FOLLOW-UP < 3 months

Study	Year	Rehabilitation group Total	Mean	SD	Control group Total	Mean	SD	Weight	MD	95% CI
Bellardinelli 2006	2006	15	2.10	0.7000	12	-0.30	0.8000	31.8%	2.40	[1.83; 2.97]
Berg 2015	2015	99	2.03	8.4800	97	-0.09	7.9500	12.1%	2.12	[-0.18; 4.42]
Nyman 2023	2023	28	1.67	6.9400	28	-0.05	6.7100	6.3%	1.72	[-1.86; 5.30]
Piccini 2013	2013	452	0.69	5.4100	400	0.11	5.2600	30.0%	0.58	[-0.14; 1.30]
Piotrowicz 2015	2015	75	2.30	4.0500	32	-0.20	3.3500	19.8%	2.50	[1.02; 3.98]
**Total (95% CI)**		**669**			**569**			**100.0%**	**1.80**	**[0.81; 2.79]**

Heterogeneity: $Tau^2 = 0.7133$; $Chi^2 = 16.21$, df = 4 (P = 0.0027); $I^2 = 75.3\%$
Test for overall effect: Z = 3.57 (P < 0.001)

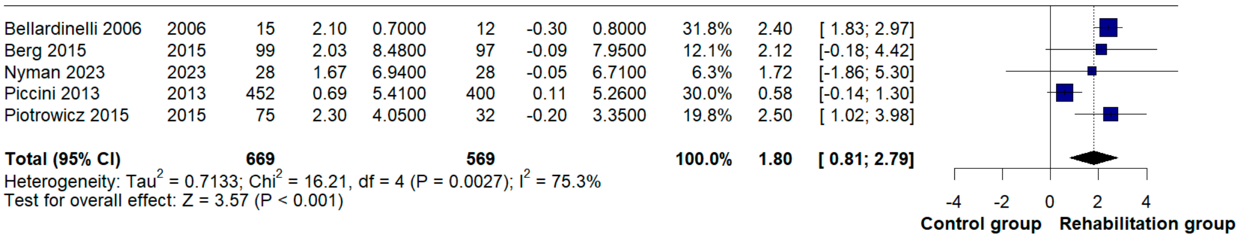

Figure S1. Forest plots of peak VO2 change at less or more than 3 months follow-up.

A. DIRECTLY SUPERVISED

Study	Year	Rehabilitation group Total	Mean	SD	Control group Total	Mean	SD	Weight	MD	95% CI
Bellardinelli 2006	2006	15	2.10	0.7000	12	-0.30	0.8000	33.9%	2.40	[1.83; 2.97]
Berg 2015	2015	99	2.03	8.4800	97	-0.09	7.9500	13.8%	2.12	[-0.18; 4.42]
Nyman 2023	2023	28	1.67	6.9400	28	-0.05	6.7100	7.3%	1.72	[-1.86; 5.30]
Piccini 2013	2013	452	0.69	5.4100	400	0.11	5.2600	32.2%	0.58	[-0.14; 1.30]
Smolis-Bąk 2017	2017	31	2.90	5.3800	32	-0.10	4.4000	12.8%	3.00	[0.57; 5.43]
**Total (95% CI)**		**625**			**569**			**100.0%**	**1.80**	**[0.73; 2.88]**

Heterogeneity: $Tau^2 = 0.7958$; $Chi^2 = 16.25$, df = 4 (P = 0.0027); $I^2 = 75.4\%$
Test for overall effect: Z = 3.30 (P < 0.001)

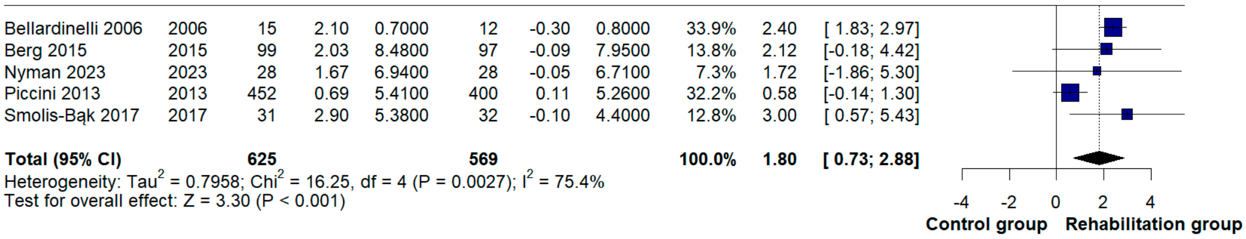

B. REMOTELY MONITORED

Study	Year	Rehabilitation group Total	Mean	SD	Control group Total	Mean	SD	Weight	MD	95% CI
Dougherty 2015	2015	84	2.30	6.9200	76	-0.10	5.9000	35.6%	2.40	[0.41; 4.39]
Piotrowicz 2015	2015	75	2.30	4.0500	32	-0.20	3.3500	64.4%	2.50	[1.02; 3.98]
**Total (95% CI)**		**159**			**108**			**100.0%**	**2.46**	**[1.28; 3.65]**

Heterogeneity: $Tau^2 = 0$; $Chi^2 = 0.01$, df = 1 (P = 0.9369); $I^2 = 0.0\%$
Test for overall effect: Z = 4.07 (P < 0.001)

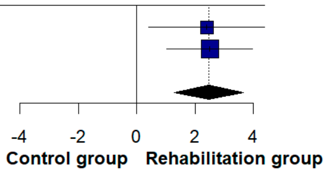

Figure S2. Forest plots of peak VO2 change in directly supervised or remotely monitored cardiac rehabilitation

A. ICD

Study	Year	Rehabilitation group Total	Mean	SD	Control group Total	Mean	SD	Weight	MD	95% CI
Belardinelli 2006	2006	15	2.10	0.7000	12	-0.30	0.8000	83.2%	2.40	[1.83; 2.97]
Berg 2015	2015	99	2.03	8.4800	97	-0.09	7.9500	5.2%	2.12	[-0.18; 4.42]
Dougherty 2015	2015	84	2.30	6.9200	76	-0.10	5.9000	7.0%	2.40	[0.41; 4.39]
Smolis-Bąk 2017	2017	31	2.90	5.3800	32	-0.10	4.4000	4.7%	3.00	[0.57; 5.43]
**Total (95% CI)**		**229**			**217**			**100.0%**	**2.41**	**[1.89; 2.94]**

Heterogeneity: $Tau^2 = 0$; $Chi^2 = 0.29$, df = 3 (P = 0.9622); $I^2 = 0.0\%$
Test for overall effect: Z = 9.02 (P < 0.001)

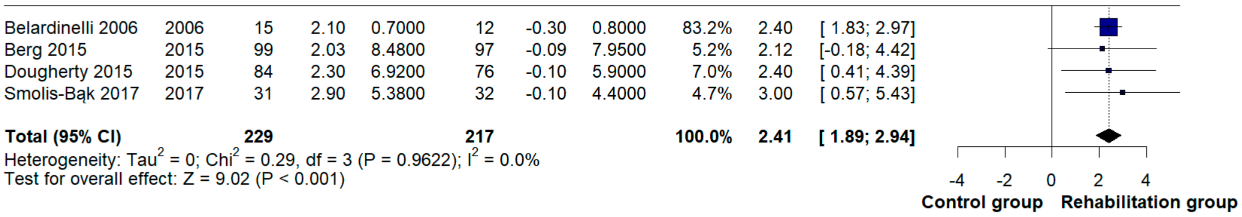

B. ICD + CRT-D

Study	Year	Rehabilitation group Total	Mean	SD	Control group Total	Mean	SD	Weight	MD	95% CI
Nyman 2023	2023	28	1.67	6.9400	28	-0.05	6.7100	13.1%	1.72	[-1.86; 5.30]
Piccini 2013	2013	452	0.69	5.4100	400	0.11	5.2600	50.5%	0.58	[-0.14; 1.30]
Piotrowicz 2015	2015	75	2.30	4.0500	32	-0.20	3.3500	36.4%	2.50	[1.02; 3.98]
**Total (95% CI)**		**555**			**460**			**100.0%**	**1.43**	**[-0.05; 2.90]**

Heterogeneity: $Tau^2 = 0.9866$; $Chi^2 = 5.42$, df = 2 (P = 0.0667); $I^2 = 63.1\%$
Test for overall effect: Z = 1.90 (P = 0.058)

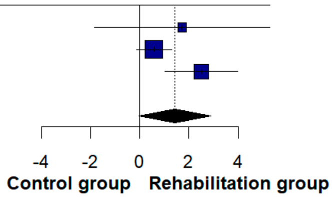

Figure S3. Forest plots of subgroups analysis regarding peak VO2 change in only ICD patients or mixed populations

A. Baseline peak VO2 > 20

Study	Year	Rehabilitation group Total	Mean	SD	Control group Total	Mean	SD	Weight	MD	95% CI
Berg 2015	2015	99	2.03	8.4800	97	-0.09	7.9500	36.3%	2.12	[-0.18; 4.42]
Dougherty 2015	2015	84	2.30	6.9200	76	-0.10	5.9000	48.7%	2.40	[0.41; 4.39]
Nyman 2023	2023	28	1.67	6.9400	28	-0.05	6.7100	15.0%	1.72	[-1.86; 5.30]
**Total (95% CI)**		**211**			**201**			**100.0%**	**2.20**	**[0.81; 3.58]**

Heterogeneity: $Tau^2 = 0$; $Chi^2 = 0.11$, df = 2 (P = 0.9452); $I^2 = 0.0\%$
Test for overall effect: Z = 3.10 (P = 0.002)

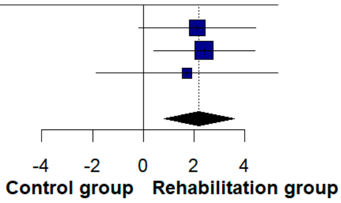

B. Baseline peak VO2 < 20

Study	Year	Rehabilitation group Total	Mean	SD	Control group Total	Mean	SD	Weight	MD	95% CI
Belardinelli 2006	2006	15	2.10	0.7000	12	-0.30	0.8000	32.9%	2.40	[1.83; 2.97]
Piccini 2013	2013	452	0.69	5.4100	400	0.11	5.2600	31.4%	0.58	[-0.14; 1.30]
Piotrowicz 2015	2015	75	2.30	4.0500	32	-0.20	3.3500	22.2%	2.50	[1.02; 3.98]
Smolis-Bąk 2017	2017	31	2.90	5.3800	32	-0.10	4.4000	13.5%	3.00	[0.57; 5.43]
**Total (95% CI)**		**573**			**476**			**100.0%**	**1.93**	**[0.80; 3.06]**

Heterogeneity: $Tau^2 = 0.9218$; $Chi^2 = 17.10$, df = 3 (P = 0.0007); $I^2 = 82.5\%$
Test for overall effect: Z = 3.35 (P < 0.001)

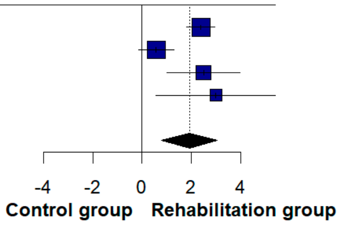

Figure S4. Forest plots of subgroups analysis regarding baseline peak VO2

A. Ischemic > 60%

Study	Year	Rehabilitation group Total	Mean	SD	Control group Total	Mean	SD	Weight	MD	95% CI
Belardinelli 2006	2006	15	2.10	0.7000	12	-0.30	0.8000	31.3%	2.40	[1.83; 2.97]
Nyman 2023	2023	28	1.67	6.9400	28	-0.05	6.7100	6.8%	1.72	[-1.86; 5.30]
Piccini 2013	2013	452	0.69	5.4100	400	0.11	5.2600	29.7%	0.58	[-0.14; 1.30]
Piotrowicz 2015	2015	75	2.30	4.0500	32	-0.20	3.3500	20.3%	2.50	[1.02; 3.98]
Smolis-Bąk 2017	2017	31	2.90	5.3800	32	-0.10	4.4000	11.9%	3.00	[0.57; 5.43]
**Total (95% CI)**		**601**			**504**			**100.0%**	**1.91**	**[0.87; 2.94]**

Heterogeneity: $Tau^2 = 0.8073$; $Chi^2 = 17.10$, df = 4 (P = 0.0019); $I^2 = 76.6\%$
Test for overall effect: Z = 3.60 (P < 0.001)

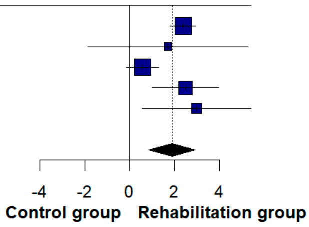

B. Ischemic < 60%

Study	Year	Rehabilitation group Total	Mean	SD	Control group Total	Mean	SD	Weight	MD	95% CI
Berg 2015	2015	99	2.03	8.4800	97	-0.09	7.9500	42.7%	2.12	[-0.18; 4.42]
Dougherty 2015	2015	84	2.30	6.9200	76	-0.10	5.9000	57.3%	2.40	[0.41; 4.39]
**Total (95% CI)**		**183**			**173**			**100.0%**	**2.28**	**[0.78; 3.78]**

Heterogeneity: $Tau^2 = 0$; $Chi^2 = 0.03$, df = 1 (P = 0.8568); $I^2 = 0.0\%$
Test for overall effect: Z = 2.97 (P = 0.003)

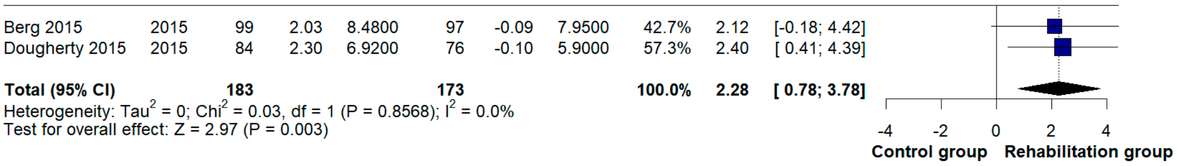

Figure S5. Forest plots of subgroup analysis regarding baseline peak VO2 and ischemic etiology

Funnel plot

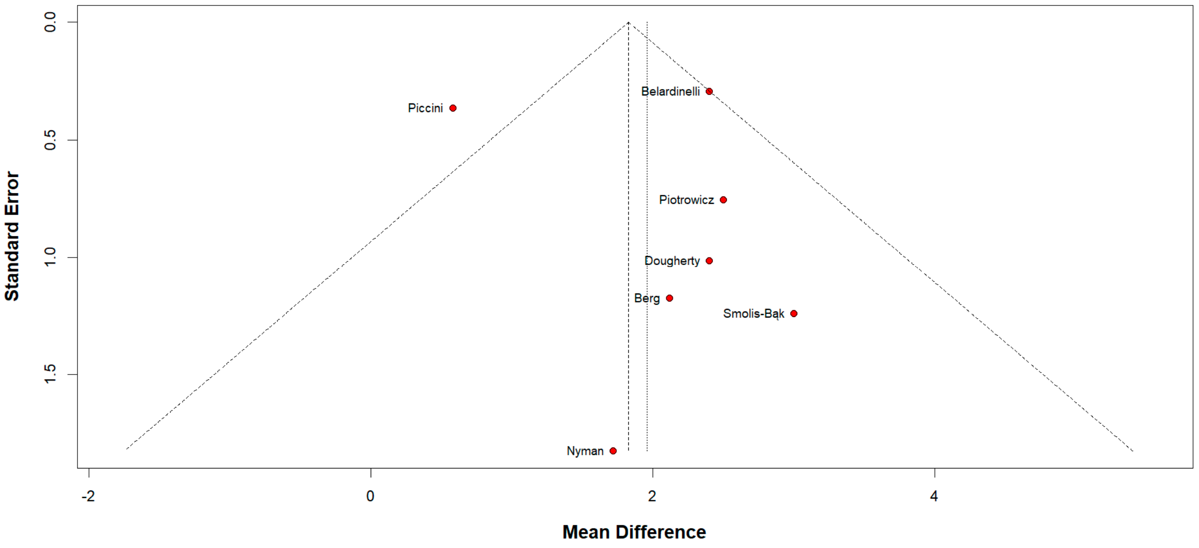

Eggers' test of the intercept

============================

intercept 95% CI t p

0.52 -1.93 - 2.97 0.416 0.6949164

Eggers' test does not indicate the presence of funnel plot asymmetry.

Figure S6. Publication Bias and Eggers' test

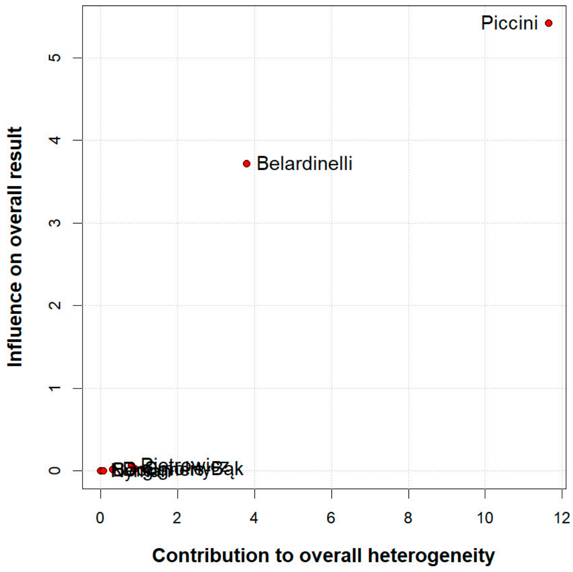

Figure S7. Baujat plot

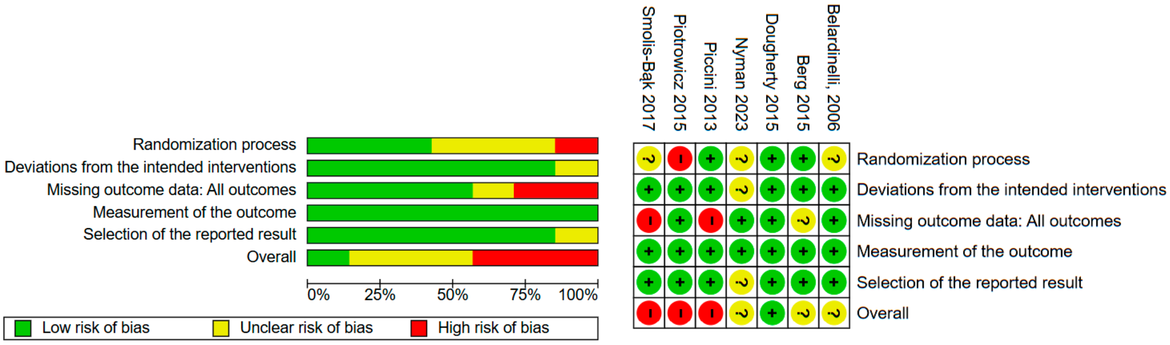

Figure S8. Risk of bias graph and summary
